# Why dentists don’t use rubber dam during endodontics and how to promote its usage?

**DOI:** 10.1186/s12903-016-0175-2

**Published:** 2016-02-25

**Authors:** Ahmad A. Madarati

**Affiliations:** College of Dentistry, Taibah University, PO Box 30034, Madina, 41477 Saudi Arabia

**Keywords:** Endodontics, Isolation, Questionnaire, Rubber dam, Root canal, Survey, Education, Alternatives

## Abstract

**Background:**

This survey study aimed at investigating the frequency of rubber dam use during root canal treatment, identifying influencing factors for not using it by Saudi general dental practitioners (GDPs) and endodontists. It also aimed at identifying measures that increase rubber dam usage.

**Methods:**

After obtaining an ethical approval, two pilot studies were conducted on staff members at Taibah University College of Dentistry and a group of GDPs. A final online survey was constructed comprising 17 close-ended questions divided into six categories: demographics, endodontic practice, rubber dam use, alternative isolation methods, reasons for not using rubber dam, and measures and policies that increase its usage. The survey was emailed to 375 GDPs randomly selected from the dental register and all endodontists (*n* = 53) working in the western province, Saudi Arabia. Data were analyzed using the Chi-square and Linear-by-Linear association tests at *p* ≤ 0.05.

**Results:**

The proportion of endodontists who used rubber dam (84.8 %) was significantly greater than that of GDPs (21.6 %) (*p* < *0.001*). Significantly the highest proportion (40.5 %) did not use rubber dam because of *unavailability* at working place. Most rubber dam none-users (69.25 %) used *a combination* of *other isolation me*ans. The highest proportion of those who used rubber dam were working in the *governmental sector* (54.3 %). Among rubber dam users, the greatest proportion graduated from Saudi Arabia (57.8 %) compared to those graduated from Egypt (34.3 %) and Syria (22.4 %). There was a significant correlation between the patterns of rubber dam use *during undergraduate* training and its usage after graduation (*p* = *0.001*). The highest proportion of participants (48.1 %) reported *better undergraduate education* as the most important factor that would increase rubber dam use in dental practice.

**Conclusions:**

Using of rubber dam was not common in Saudi general dental practice. Dentists must follow the recommended standards of care. Place of work and patterns of using rubber dam during undergraduate study were the most influencing factors. Better undergraduate education was the most important proposed measure to increase its usage. The combination of cotton rolls and saliva high-volume ejector or gauze was the most common alternative to rubber dam isolation.

## Background

Although the concept of isolating teeth undergoing root canal treatment (RCT) was first introduced 150 years ago [1], to this date, rubber dam (RD) is still the ideal tool for tooth isolation during dental therapeutic procedures. It has several advantages during RCTs for dental professionals and patients. It facilitates washing and scrubbing the working field and prevents salivary contamination; hence it enables the preparation of an aseptic working field. RD also, helps protecting patients from inhalation or ingestion of endodontic instruments, retracting soft tissues, and contributing to efficient treatment [2–4]. Consequently, RD isolation during RCTs has been considered as a standard of care [2]. A previous questionnaire study showed that 75 % of respondents felt that RD should be used compulsory during RCTs [5]. This was in agreement with Heling & Heling who reported a case of swallowing of endodontic files [6], which may cause patients’ death [7]. However, reports have shown lack of RD use among clinicians in several countries [8, 9] with only a few exceptions [10, 11]. Many reasons were reported such as: placement difficulty, time consumption, patients’ rejection, lack or insufficient training, and high cost [3, 9, 12–14]. In addition, gender, undergraduate and postgraduate training, treated tooth and number of RCTs performed, year of qualification, graduation from different schools, practice location and type, and high interest in endodontics have been investigated as possible influencing factors [8, 9, 12, 14–19].

Many questionnaire studies were conducted in different countries and reported various usage frequencies. Yet in some countries, like the United Kingdom and the United States of America, different studies were conducted at different periods of time; showing the trend of RD usage over time [8–10, 12, 15–17, 19–21]. This may give an insight to attitudes and preferences of practitioners towards RD usage over different times. However, conducting studies in different countries may reveal differences in practices and preferences among practitioners of these countries. This can reflect, to certain extent, the impact of professional environment, undergraduate and postgraduate curricula, educational guidelines and governmental regulations on attitudes and preferences of clinicians in a specific country. Ahmed et al. stressed the importance of further research, especially on educational methods, to overcome the discrepancy between the well adoption of RD during undergraduate training and the low frequency of usage after graduation [22].

Within this respect, a single study inspected the frequency of RD use in Saudi Arabia. It did not investigate the different aspects of RD use as the questionnaire was about general RCTs procedures [23]. Therefore, conducting a questionnaire study will give insight into attitudes and preferences of dental clinicians, practicing in Saudi Arabia, towards using RD during RCTs. The survey study was divided into two parts. This part of the study aimed at investigating the frequency of RD use during RCT and its influencing factors. It also aimed at identifying measures that increase its usage. Therefore, the null hypotheses of this study were:

I. There would be no significant differences among respondents regarding the reasons for not using RD isolation during RCTs.
II. There would no significant differences among respondents in reporting factors that may increase RD use in dental practice.

## Methods

This survey study was conducted in accordance with the World Medical Association Declaration of Helsinki (version 2013). The study, including the questionnaire form, was ethically approved by the Research Ethics Committee (REC) at College of Dentistry, Taibah University, Saudi Arabia. Participants’ personal information including email addresses remain confidential in web-based surveys, therefore the ethical committee approved this study without the need for obtaining a consent form from each participant. The methodology was that described in another part of the study (accepted for publication). Briefly, a sample size calculation was carried out through population for descriptive sampling technique with an expected response rate between 40 and 60 and 90 % power calculation. Hence, 375 GDPs were randomly selected by a third person who was not related to the study. A first pilot self-administrated questionnaire was distributed to the academic staff members at College of Dentistry, Taibah University to formulate a questionnaire that includes relative aspects. A second pilot study was conducted on a sample of general dental practitioners (GDPs) to ensure that questions were easily understood. A final online questionnaire was constructed using the Google Drive tool. The web-based questionnaire related to this part comprised 17 close-ended questions in five categories; a) demographics, b) general endodontic practice, c) usage of RD, d) reasons for no use, e) alternative methods for tooth isolation during RCT, and f) policies and measures to increase RD usage. The questionnaire was electronically sent to the 375 selected GDPs and all endodontists working in the western province, Saudi Arabia (53). The email explained study aims and confirmed that participants’ identity would remain anonymous. A reminder email was sent to all selected dentists and endodontists after 8 weeks. Responses were collected and data were entered into SPSS 19 for Windows software (SPSS Inc., Chicago, IL, USA). Data were analyzed using the Chi-square and Linear-by-Linear association tests at the *0.05* level of significance.

## Results

### Classification of respondents

Of the 237 who responded to this study, 175 (73.8 %) were GDPs, 34 (14.3 %) endodontists, 9 (3.8 %) students or residents in endodontic postgraduate studies programmes, and 19 (8 %) others (residents in other field such as orthodontics or periodontics, but they were performing root canal treatments). It is well known, in Saudi Arabia, that some specialists perform dental treatments, including RCTs, in addition to their specialized treatments. Placing RD is mandatory during endodontic postgraduate studies programmes in Saudi Arabia; hence students or residents in endodontic postgraduate studies programmes were classified as endodontists for specific variable for better statistical analysis.

### Response rates

The total number of sample size was 428 (375 GDPs and 53 endodontists). The overall response rate was 237 / 428 = 55.4 %. GDPs and *others*’ response rate was: 203 / 375 = 54.1 %. Response rate for endodontists was: 34 / 53 = 64.2 %.

### Non-response bias

There was no significant difference between the proportion of *early respondents* who used RD for RCTs (36.4 %) and that of *late respondents* who did so (39.7 %) [*p* = 0.681].

### Usage of RD

Overall, a significantly greater proportion of respondents (62.7 %) did not use RD (*p* < 0.001) (Table 1). The proportion of endodontists who used RD (84.8 %) was significantly greater than that of GDPs (21.6 %) [*p* < 0.001].

**Table 1:** Frequency of rubber dam use

	Use of rubber dam (%)		
Respondents	Yes	No	Total
Endodontists	28 (84.8 %)	5 (15.2 %)	33 (100 %)
Endodontics Postgraduate Students	9 (100 %)	0 (0 %)	9 (100 %)
GDPs	33 (21.6 %)	120 (78.4 %)	153 (100 %)
Other	8 (57.1 %)	6 (42.9 %)	14 (100 %)
Total	78 (37.3 %)	131 (62.7 %)	209 (100 %)

### Reasons for not using RD

All those who did not use RD reported the reasons for doing so (Table 2). Significantly, the highest proportion of them (40.5 %) did not use RD because it was *not available* at the working place (*p* < 0.001). Overall, there were no significant differences between endodontists and GDPs (*p* = 0.265).

**Table 2:** Reasons for not using rubber dam (%)

Respondents	Difficult use	Time consuming	Not available at work	Others	Total
Endodontists	0	0	80	20	100
GDPs	23.3	16.7	39.2	20.8	100
Others	0	16.7	33.3	50	100
Total	21.4	16	40.5	22.1	100

### Alternatives of RD isolation

Overall, there were no significant differences between endodontists and GDPs (*p* = 0.512). Significantly, the highest proportion of RD none-users (69.25 %) used a combination of at least two of *other isolation me*ans (*cotton roll*, *saliva ejector* or *throat pack*) [*p* < 0.001] (Table 3).

**Table 3:** Alternative methods for isolating teeth receiving RCTs (%)

Respondents	No need for isolation	Combination of at least two of other means	Cotton roll	Suction	Total
Endodontists	0	100	0	0	100 (5)
GDPs	0.8 (1)	68.1 (81)	26.1 (31)	5 (6)	100 (120)
Others	0 (0)	66.4 (4)	33.3 (2)	0 (0)	100 (6)
Total	0.8 (1)	69.2 (90)	25.4 (33)	4.6 (6)	100 (130)
Early Respondents	1.2 (1)	69.9 (58)	24.1 (20)	4.8 (4)	100 (83)
Late Respondents	0 % (0)	68.1 (32)	27.7 (13)	4.3 (2)	100 (47)

The numbers in parentheses are frequencies of respondents

### Experience of participants

Overall, there was no significant difference between the proportions of the four categories of *respondents’ experience* (*p* = 0.480) (Table 4). However, whilst the highest proportion of GDPs (32.8 %) had *up to 3 years*’ experience, the highest proportion of endodontists (58.8 %) had *7.1 to 15 years’* experience (*p* < 0.001). There was no significant correlation between *respondents experience* and using RD (*p* = 0.844).

**Table 4:** Respondents details regarding years of practice after graduation (experience) (%)

Respondents	Up to 3 years	3.1 to 7 years	7.1 to 15 years	More than 15 years	Total
GDPs	32.8 (42.4)	27 (33.3)	20.1 (15.2)	20.1 (9.1)	100 (21.6)
Endodontists	0	11.8 (14.3)	58.8 (60.7)	29.4 (25)	100 (84.8)
Endo Postgraduate students	11.1 (11.1)	77.8 (77.8)	11.1 (11.1)	0 (0)	100 (100)
Others	10.5 (12.5)	21.1 (12.5)	47.4 (62.5)	21.1 (12.5)	100 (57.1)
Total	25.4 (20.5)	26.3 (29.5)	27.5 (35.9)	20.8 (14.1)	100 (37.3)

The values in parentheses represent proportion of respondents who currently use RD

### Number of RCTs per week

Nearly 12 % of participants never performed RCTs (Table 5). Whilst the highest proportion of endodontists (45.2 %) performed *more than 12 RCTs per week,* only 26.1 % of GDPs did so (*p* = 0.002). The number of RCTs significantly and linearly correlated with participants’ experience (*p* < 0.001) (Table 6). There was no linear correlation between the *number ofRCTs* and the use of RD (*p* = 0.400).

**Table 5:** Number of RCTs performed per week (%)

Respondents	Never do RCTs	1–2 cases	3–5 cases	6–10 cases	More than 12 cases	Total ^a^
Endodontists	2.3	2.4	21.4	31	45.2	100
GDPs	12.6	22.2	28.1	23.5	26.1	100
Other	26.3	50	28.6	7.1	14.3	100
Total	11.8	20.1 (50)	26.8 (30.4)	23.9 (34)	29.2 (37.7)	100 (37.3)

^a^The total of those who were performing RCTs only. The values in the brackets represent proportions of RD users within *Number of RCT cases performed per week* groups

**Table 6:** Correlation of weekly performed RCTs and respondents’ experience (%)

Respondents’ experience (years)	1–2 cases	3–5 cases	6–10 cases	More than 10 cases	Total
Up to 3	40	26	20	14	100
3.1 to 7	16.4	32.7	21.8	29.1	100
7.1 to 15	17.5	25.4	20.6	36.5	100
More than 15	4.9	22	36.6	36.5	100
Total	20.1	26.8	23.9	29.2	100

### Type of work

The proportion of those who were working in *private* (51.5 %) was significantly greater than that of governmental sector (40.3) [*p* = *0.038*] (Table 7). Whilst the highest proportion of GDPs (60.8 %) worked in *private* sector, the highest proportion of endodontists (48.5 %) worked for *government* (*p* < 0.001). Whereas, the majority of those who were working in *private sector* did not use RD (85.2 %), the higher proportion of those who were working in the *academic* (90 %) and *governmental* sectors (53.7 %) used it (*p* < *0.001*).

**Table 7:** Rubber dam use according to type (place) of work (%)

Respondents	Private	Academic	Government	Postgraduate Programme	Total
GDP	60.8 (9.7)	0.6	38.3 (37.9)	0 (0)	100 (21.6)
Endodontists	27.3 (55.6)	24.2 (100)	48.5 (93.8)	0 (0)	100 (84.8)
Endo Postgraduate students	0 (0)	0 (0)	37.5 (100)	62.5 (100)	100 (100)
Other	31.6 (33.3)	21.1 (66.7)	42.1 (80)	5.3 (0)	100 (57.1)
Total	51.5 (14.8)	5.6 (90)	40.3 (53.7)	2.6 (100)	100 (37.3)

The values in parentheses represent proportion of respondents who currently use RD

### Country of bachelor degree

Significantly, the highest proportion graduated from *Saudi Arabia* and *Syria* (29.8 and 22.4 %, respectively) (*p* < 0.001) (Table 8). The proportion of *Saudi Arabia* graduates who used RD (56.8 %, excluding postgraduate students) was significantly greater than that of *Egypt* and *Syria* graduates and used RD (35.3 and 20.8 %, respectively) [*p* *= 0.005*]. The highest proportion of those who graduated from *Saudi Arabia* were working in *governmental sector* (68.4 %), which was significantly greater than those who graduated from *Syria, Jordan and Egypt* and worked in the same sector (18, 43.8 and 52.8 %, respectively) [*p* *< 0.001*].

**Table 8:** Rubber dam use according to country of bachelor degree and place of work (%)

Place of work	Saudi Arabia	Syria	Jordan	Egypt	Other Arab Countries	Other Countries
Private	24.6 [33.3]	80 [10.5]	50 [28.6]	41.7 [6.7]	73.3 [15]	58.3 [15.4]
Academia	7 [75]	2 [100]	6.2 [100]	5.6 [90]	6.7 [100]	12.5 [100]
Government	68.4 [61.3]	18 [55.6]	43.8 [57.1]	52.8 [57.9]	20 [33.3]	29.2 [28.6]
Total	100 [56.8]	100 [20.8]	100 [46.7]	100 [35.3]	100 [25]	100 [27.3]
	(29.8)	(22.4)	(7)	(16.2)	(13.2)	(11.4)
	100 [37.3]					

The values in brackets represent proportion of respondents who used RD within each corresponding country group

### Training on RD use

Significantly, the highest proportion (67.4 %) *did not have training* on RD placement (*p* < 0.001) (Table 9). The proportion of participants who *had extensive training* and used RD (71.4 %) was significantly greater than those who *did not have* or had *little* training and used RD (35.5 and 26.5 %, respectively) [*p* *= 0.001*].

**Table 9:** Rubber dam use according to country of graduation and previous training on RD placement (%)

	Country of bachelor degree					
Training on RD use	Saudi Arabia	Syria	Jordan	Egypt	Other Countries	Total
No	76.5 (58.3)	62.7 (20)	81.2 (46.2)	70.3 (29.2)	54.5 (24.1)	67.4 (35.5)
Extensive	11.8 (100)	7.8 (50)	0 (0)	13.5 (60)	9.1 (60)	9.3 (71.4)
Little	11.8 (33.3)	29.4 (20)	18.8 (50)	16.2 (33.3)	36.4 (23.5)	23.3 (26.5)
Total	100	100	100	100	100	100 (37.3)

The values in parentheses represent proportion of respondents who used RD

### RD use during undergraduate study

Overall, significantly the highest proportion (38 %) reported *mandatory RD use for all RCTs steps including access cavity* during undergraduate study (p *<0.001*) (Table 10). Overall, there was a significant positive correlation between the *use of RD during undergraduate* study and using RD after graduation (*p* = *0.001*). The proportion of participants who graduated from *Saudi Arabia* and reported a *mandatory RD usage during all RCTs procedures* (majority 79.4 %) was significantly greater than that graduated from *Jordan* and reported the same usage pattern (43.8 %) [*p* = *0.048*].

**Table 10:** Rubber dam use according to country of graduation and usage pattern during undergraduate study (%)

	Country of bachelor degree				
Strategy of RD use during undergraduate study	Saudi Arabia	Syria	Jordan	Egypt	Total ^a^
Not in the curriculum	0 (0)	54.9 (36.4)	0 (0)	18.9 (8.3)	20.7 (22.2)
Optional	1.5 (3.8)	33.3 (36.4)	6.3 (14.3)	24.3 (33.3)	19 (31)
Mandatory during one stage	1.5 (0)	3.9 (9.1)	6.3 (14.3)	2.7 (0)	4.2 (22.2)
Mandatory during one RCT	1.5 (0)	2 (0)	0 (0)	8.1 (8.3)	3.4 (28.6)
Mandatory for all RCTs after Access cavity	16.2 (19.2)	3.9 (9.1)	43.8 (42.9)	29.7 (25)	14.8 (41.9)
Mandatory for all RCTs including access cavity	79.4 (76.9)	2 (9.1)	43.8 (28.6)	16.2 (25)	38 (50.7)
Total	100	100	100	100	100 (37.3)

^a^The total proportions were calculated out of all those who responded to this questions. The values in parentheses represent proportion of respondents who used RD

### Policy for RD usage at different dental practice

#### *Policy for undergraduate study training use*

The majority (91.1 %) recommended *mandatory* use of RD during *undergraduate training* (*p* < 0.001) with no significant difference between endodontists and GDPs (*p* = *0.472*) (Table 11).

**Table 11:** Respondents’ recommended policy for RD usage during undergraduate study and general and endodontic specialized practice (%)

	During undergraduate study		General dental practice		Endodontic specialized practice	
Respondents	Optional	Mandatory	Optional	Mandatory	Optional	Mandatory
Endodontists	4.7	95.3	9.3	90.7	0	100
GDPs	9.8	90.2	25.9	74.1	11.7	88.3
Other	11.1	88.9	31.6	68.4	12	88
Total	8.9	91.1	23.3	76.7	9.5	90.5

#### *Policy for general dental practice use*

Significantly, the highest proportion (76.7 %) suggested *Mandatory* use of RD in *general dental practice* (*p* < 0.001) (Table 11); with a significantly greater proportion of endodontists compared to that of GDPs (90.7 and 74.1 %) [*p* = *0.048*].

#### *Policy for endodontic specialized practice use*

The majority (90.5 %) suggested *mandatory* use of RD in *endodontic specialized practice* (*p* < 0.001) (Table 11); with no significant difference between endodontists and GDPs (100 and 88.3 %) [*p* =* 0.063*].

### Factors may contribute to better RD usage

Significantly, the highest proportion of participants (48.1 %) believed that *better undergraduate education* is the most important factor that contributes to better implementation of RD in dental practice followed by *strict governmental regulations* (22.8 %) [*p* < *0.001*] (Table 12). There was no significant difference between endodontists and GDPs (*p* =* 0.066*).

**Table 12:** Measures that may increase RD use in dental practice (%)

Respondents	Better undergraduate education	Patient’s education	Strict governmental laws	More post-graduate training	Increase treatment fees	Total
Endodontists	60.5	0	27.9	2.3	9.3	100
GDPs	42.9	9.1	21.1	16.6	10.3	100
Other	68.4	5.3	26.3	0	0	100
Total	48.1	7.2	22.8	12.7	9.3	100

## Discussion

Questionnaires reporting attributes, preferences, practices and demographies of participants are an important research tool. However, they should be well conducted to enable high response rates, therefore the results can be generalised [24]. This study was conducted on GDPs and endodontists of the western province, Saudi Arabia only; which can be considered as a limitation. However, our unpublished data revealed no significant differences in endodontic practice among the different provinces of Saudi Arabia. Well identification of practice’s aspects and correct questions’ formats are important features of survey studies. However, good sampling and none-response bias are essential factors [24]. Two pilot studies on staff members at College of Dentistry, Taibah University and a group of GDPs were conducted to obtain easily answered survey which in turn eliminates interpretation-related bias [24]. A high response rate (70–80 %) is preferred to minimize the risk of bias [25]. However, a response rate as low as 43 % has the least none-response bias [26]. Hence, the overall 55.4 % response rate obtained in this study is satisfactory. This is especially true with the fact that internet-based surveys achieve lower response rates than those of postal ones [27]. Approaching dentists by email rather than mail post service can be another limitation of this study. Results of survey studies can be invalid if the none-respondents differ from the respondents [28]. Also, if the none-response is not due to questionnaire design, then the none-respondents can be ignored and the respondents can be used as a representative sample of the population [28]. One accepted method to investigate none-response bias is to determine the late-response bias by comparing responses of participants who responded to the survey after the first request, with those who responded after reminder requests [29]. The results of our study did not reveal non-response bias; in that there was no significant difference between the proportion of *early respondents* who used RD for RCTs (36.4 %) and that of *late respondents* who did so (39.7 %).

RD isolation of teeth undergoing RCTs has been considered mandatory and standard of care due to its advantages as discussed earlier [2]. Nevertheless, our results showed that only 21.6 % of GDPs were using RD. This poor usage rate, however, is not an exception and is within the rates’ range reported in several previous questionnaires; 2 % [18], 12 % [30], 20 % [31] and 30 % [19]. However, there has been obvious increase in RD usage among Saudi dental practitioners compared to previous studies [23, 32]. This could be attributed to the current undergraduate curricula compelling mandatory RD use during RCTs procedures. Also, these findings may reflect improvement in clinician’s awareness towards the importance of using RD. Previous studies, conducted in UK and USA, reported similar improvement of RD usage over a period of time [33, 34].

A variety of disincentives to regular RD use amongst GDPs have been suggested. One report showed that the most common causes for GDPs negative attitudes were “*inconvenience*” and that “RD was *unnecessary*” [10]. Such poor decision-making in clinical practice may reflect GDPs’ need of compiling strong evidence to change their attitude towards RD application. Unfortunately, clinical cohort studies with a control group of patients receiving root canal treatments without RD is unethical. Nevertheless, a recent study reported a significantly greater survival probability of teeth when an initial RCT was performed using RD (90.3 %) than the 88.8 % observed among those treated without RD [35]. The most commonly reported reason for not using RD, in the current study, was its *unavailability* at working place (40.5 %). Interestingly, 80 % of endodontists who were not using RD and reported this reason were working in private practice. This stress the need for more attention and strict regulations to be applied in private sector and regularly monitored. This is especially true as it is well known among endodontists that using RD is a standard of care. Nevertheless, it can be speculated that reporting such a reason may reflect the willingness of practitioners to use RD once available. The second and third most common reasons were *difficult placement* (21.4 %) and *time consuming* (16 %), which were also reported in previous studies [9, 10, 33, 36, 37]. Application of RD during RCTs is mandatory for undergraduate students in almost all Saudi dental institutes. Hence, attributing poor usage to *difficult placement* is invalid. Indeed, some clinicians considered the ability to place RD successfully can be achieved by experience, which, in turn can only be attained by regular use [13, 37]. The difficulty in taking radiographs with the dam in-place, especially for working length or cone fit determination, is another possible reason for not using RD [38]. However, prevention of swallowing or inhalation of endodontic instruments and materials should be a great incentive. Similarly, claiming that placing RD is a *time consuming* procedure (16 %) is unacceptable; as RD can be placed, even by inexperienced dentists, in few minutes (1–8 min) [13, 39–42]. Time required for placing RD is perceived by some clinicians, unfortunately, as wasted time rather than time spent on an essential procedure for a successful and safe RCT. Nevertheless, this time is far less than the time required for changing cotton rolls and frequent rinsing by the patient [41, 43]. Also, time saved by working in a clean and aseptic field with good visibility may compensate for the time spent for RD placement [43]. Like other reports [9, 10, 12, 33, 37], 7 % of the current study respondents claimed that they did not use RD because of *patients’ discomfort* or *patients’* rejection. However, reports showed that patients have no objection, and moreover, they would prefer RD placement in future visits [39–42]. In fact, patients’ negative attitudes, when exists, can be usually explained in light of lack of experience, competency, enthusiasm and communication skills of the clinicians and assistants. Reports have shown that clinicians’ positive attitudes [40] and their enhanced experience [41, 42] can increase patients’ acceptance.

Those who were not using RD reported other isolation methods including: cotton roll, high-volume saliva ejector or throat pack; with the highest proportion of them (69.25 %) using a combination of two of these methods. Previous surveys showed that the majority of those who did not use RD (83–88 %) were using cotton rolls alone or with other isolation means [18, 19, 34]. These alternative methods may protect the cheeks, lips, tongue and intra-oral soft tissue. However, their efficacy in providing complete isolation, especially against liquids that irritate soft tissues, is questionable. They, also, cannot prevent accidental ingestion or inhalation of endodontic instruments and materials; which can be life-threating accidents [6, 7]. It could be argue that ingestion or inhalation of endodontic instruments can be prevented by securing them by dental floss or by using rotary files mounted on a hand-piece. However, these methods are less satisfactory than RD in respect of providing aseptic field [17].

As expected, the proportion of endodontists who were using rubber dam (84.8 %) was significantly greater than that of GDPs (21.6 %). These findings were consistent with those obtained in a study conducted in USA [42]. This could be explained by the advanced training on RD placement that endodontists gain during postgraduate study programmes. Better training contributes to more RD usage [8]. The deeper understanding of its use advantages is another possible reason. Moreover, endodontists may become more conscious about the risks their patients might be exposed to without RD placement. Performing RCTs using RD is mandatory during postgraduate and residency programmes, which gradually improves postgraduate students and residents’ skills in its use. The more they use RD, the easier and quicker they can place it. This in turn, may attribute, to great extent, to better attitudes towards RD placement when working as endodontists independently. Within this respect, some of the reasons that GDPs reported for their negative attitude such as *difficult placement* and *time consumption* [36, 37] can be related indirectly to the infrequent usage.

Results showed no significant differences among the four categories of participants’ experience after graduation regarding RD usage; which were consistent with those of previous reports [12, 15, 34, 43, 44]. RD usage is usually abandoned by GDPs after graduation [3, 34]. However, the highest proportion of GDPs users (42.4 %) was within the *recently graduated* group, which is in consistent with a previous study [30]. Recently graduated practitioners are probably educated and trained with advanced methods which concentrate on the increasing attention and the advantages of RD isolation. In addition, they are exposed to more recent research which may contribute to better attitude towards RD compared to old practitioners [14]. The enthusiasm of the recent graduates who want to prove themselves may motivate them to provide higher quality of dental services.

In agreement with a previous study [45], our results showed that the majority of those who did not use RD were working in *private*. On the other hand, the majority of RD users were working in the *academic* and *governmental* sectors. Saudi governmental sector includes Ministry of Health, Universities, National Guard and Military Service. Generally, governmental hospitals in Saudi Arabia are well equipped; hence RD armamentaria are readily available. In light of this assumption, the most common reason for not using RD reported in our study was *unavailability*. Also, practitioners may be motivated to follow regulations of high standards; realizing that they are being watched by their peers working at the same place. A previous study did report greater usage of RD amongst operators in *group* practice than *solo* one [8].

The popularity of RD use in governmental sector may explain its popularity among clinicians graduated from Saudi institutes. The majority of them (68.4 %) was working in the *governmental sector*; which was significantly greater than those who graduated from other countries and worked in the same sector. Undergraduate curricula of different dental schools was investigated as a possible influencing factor on RD usage [9, 12, 14, 19]. Only one study reported a significant influence of such factor when respondents graduated from two different schools were compared [12]. The majority of previous studies’ respondents qualified from universities of countries where the studies were conducted in. By contrast, only 29.8 % of the current study’ respondents qualified from Saudi Arabia. Qualifying from overseas countries of different undergraduate curricula reflected clearly on respondents’ preferences in using RD. The proportion of participants who graduated from Saudi Arabia and used RD was significantly greater than that of participants who graduated from Egypt and Syria and used RD. The more RD is used during the undergraduate study, the greater the trend towards using it after graduation. The proportion of participants graduated from Saudi Arabia and reported a *mandatory RD usage during all RCTs procedures* (majority; 79.4 %) was significantly greater than that of participants who graduated from *other countries* and reported the same *undergraduate policy of RD usage*. This stresses the importance of further research to investigate the impact of different methods of undergraduate education in better implementation of RD usage in practice after graduation [22]. Providing proper training, whether during undergraduate or postgraduate studies, is of great importance. A previous study reported that RD non-users would use it if they knew how to simply place it [12]. In addition, Joynt et al suggested that there should be greater emphasis during the educational process on explaining the importance and the reasons for using RD rather than on placement techniques [8]. Interestingly, our results showed that the majority of those who received *intensive training* were using RD [8]. These findings are in consistent with those obtained in previous reports [8, 16]. However, only 9.3 % of the current study respondents received *intensive training*. This stresses the need for better education and training on aspects of RD usage especially during undergraduate courses.

The vast majority of respondents believed that using RD should be *mandatory* during *undergraduate* training (91.1 %) and in *dental practice* after graduation; *general and specialized* (76.7 and 90.5 %, respectively). This may reflect their awareness and understanding of its advantages. However, only 37.35 of respondents (21.6 % GDPs) were using RD. Ahmed et al. stressed the importance of further research, especially on educational methods, to overcome the discrepancy between the well adoption of RD during undergraduate training and the low frequency of usage after graduation [22]. In addition these findings suggest the need for strict governmental regulations which was confirmed by our results. The second highest proportion of participants (22.8 %) reported that *strict governmental regulations* will contribute to better RD usage. However, self-motivation maybe more effective. The highest proportion of our study’s respondents (48 %) reported *better undergraduate education* as the most influencing factor. Whitworth et al. reported that academic institutions had a significant influence on RD use, which was greater in *newly-qualified graduates* in comparison with *older practitioners* [12]. It can be speculated that recently graduated practitioners received better training on RD use. Mala et al. recommended focusing on operator concerns such as difficulty of application [14]. Some may argue that patients’ rejection can be a major obstruction of placing RD and efforts should be made first on patients education. Our results showed that patients’ education is the least important factor. As discussed earlier, patients have no objection to RD application and yet they would prefer it in future visits [39–42].

## Conclusions

Within the limitations of this study, the following can be concluded:

- Rubber dam is not being used commonly in general dental practices in Saudi Arabia.
- The *combination of cotton rolls* and *saliva high-volume ejector* or *gauze* was the most common alternative to rubber dam isolation.
- *Place of work* and *patterns of using rubber-dam during undergraduate study* were the most important factors influencing rubber dam use among GDPs.
- Better *undergraduate education* can be the most effective measure to increase its usage in dental practice.

## Abbreviations

RCT: root canal treatment
RD: rubber dam
GDPs: general dental practitioners
